# Procurement, Processing, and Storage of Human Amniotic Membranes for Implantation Purposes in Non-Healing Pressure Ulcers

**DOI:** 10.3390/mps8010012

**Published:** 2025-02-01

**Authors:** Lina A. Gómez, Carlos Domínguez-Paz, Juan F. Ospina, Elga J. Vargas

**Affiliations:** 1 Biomedical Research Center (CIBUS), Faculty of Medicine, Universidad de La Sabana, Chía 53753, Colombia; lina.gomez3@unisabana.edu.co; 2 Department of Prototypes and Manufacturing, Grupo de Energia, Materiales y Ambiente (GEMA), Universidad de La Sabana, Chía 140013, Colombia; carlos.dominguez2@unisabana.edu.co; 3 Hospital Universitario La Samaritana, Ginecohus Research Group, Bogotá 111321, Colombia; 4 Pathology Laboratory, Department of Pathology, Faculty of Medicine, Universidad de La Sabana, Chía 140013, Colombia

**Keywords:** amniotic membrane, preparation, procurement, properties, regenerative medicine, tissue engineering, wound, wound care, wound healing

## Abstract

The human amniotic membrane (hAM) has been used as an implant to enhance the regenerative process and control inflammation in different diseases, given their structure, biocompatibility, presence of stem cells and multiple growth factors. The objective of this study was to generate a standardized protocol for obtaining, processing, and storing hAMs that guarantee the conservation of their structural and cellular characteristics as well as their mechanical properties, ensuring their ease of handling, sterility, and quality that allows their implementation for therapeutic purposes in the field of regenerative medicine. The hAMs were obtained from mothers with healthy, full-term, controlled pregnancies and by cesarean section. The hAMs were processed under sterile conditions, manually separated from the placenta and, subsequently, they were frozen in a solution of culture medium plus 50% *v*/*v* glycerol. The protocol allows obtaining sterile hAMs composed of both epithelium and stroma with adequate preservation of the amniotic cells. The glycerol’s impact on the mechanical properties may enhance the membrane’s adaptability and conformability to diverse wound surfaces, potentially improving the healing process. It is necessary to repeat microbiological, cell viability and mechanical studies at 6 and 12 months to ensure that long-term frozen conditions do not affect the quality of the hAMs.

## 1. Introduction

The placenta is the main site of gas and nutrient exchange between the mother and fetus. The amnion, the innermost layer, delimits a sac that is filled with amniotic fluid, which surrounds and protects the embryo and covers the umbilical cord.

The amniotic membrane is translucent, avascular and is composed of five layers: a monolayer epithelium of cuboidal cells resting on a basement membrane rich in type IV and VII collagen, fibronectin, laminin and hyaluronic acid. The third layer, called compact, is acellular and is composed of collagen types I, III, V and VII. In the fourth layer, called fibrous, fibroblasts, pluripotent stromal/mesenchymal cells and a few macrophages can be observed. The last layer, called spongiosa, has type III collagen and is rich in proteoglycans and glycoproteins, which allows the amnion to slide over the chorion [1]. The amnion, although adjacent to the chorion, is not completely fused with it, so both can be easily separated with surgical forceps or manually. At the time of delivery, the amnion has an average thickness of 0.2 to 0.5 mm [2].

Amniotic epithelial cells (AECs) are pluripotent express stem cell markers that produce growth factors (GFs) and cytokines that promote cell proliferation and differentiation [3]. Due to its biochemical composition, the human amniotic membrane (hAM) has biological and mechanical properties that allow it to be considered as a biomaterial for tissue regeneration [4].

As part of regenerative treatments, hAMs have been used in the form of a patch, locating the epithelial part in contact with the tissue alteration. This side of the membrane is rich in anti-inflammatory cytokines and GFs, such as the epidermal growth factor (EGF), vascular endothelial growth factor A (VEGF-A), keratinocyte growth factor (KGF), growth factor of hepatocyte growth factor (HGF), platelet-derived growth factor (PDGF) and fibroblast growth factor type 2 (FGF-2), also have antifibrotic properties due to the downregulation of Transforming Growth Factor Beta (TGF-β) [5].

The use of hAM implants regulates hydroelectrolyte transport and reduces pain by covering the nerve endings exposed by tissue loss. Also, HAMs express β-defenses (HBD 1, 2 and 3), localized in the amnion epithelium, decidua, and chorion trophoblast layer, which are antimicrobial peptides with antibacterial, antiviral, and antifungal activity. Elafin is a protein with antimicrobial and antiprotease functions (inhibiting proteases such as neutrophil elastases helping to control the inflammatory response). It is present in the amnion epithelium and decidua. Elafin mRNA expression significantly increases in response to the proinflammatory cytokine IL-1β and has chemoattractant properties. In this way, the hAM implants function as a protective bandage, which also reduces inflammation and improves healing [6,7].

In ocular pathologies, hAMs have been used for the treatment of epithelial defects for chemical burns, corneal abrasions ulcers, keratitis, limbal insufficiencies, band keratopathies, etc. [8,9,10]. It has also been shown that hAMs are an effective and efficient treatment for diabetic foot ulcers, pressure ulcers (PUs)—the prevalence of PS in hospitalized patients ranges from 5.3% to 26.7%, increasing care costs, days of hospital stay and care time—plantar fasciitis and skin burns [10,11,12,13,14].

In the study conducted by Berhane CC et al. in 2019 in the United States, 10 patients with PUs were treated with dehydrated human chorion and amnion membrane allografts, applied weekly along with standard care. After the first application, 70% of the PUs were reduced in size. At 2 weeks, 40% of the ulcers had reduced in size by 50%, and at 4 weeks, 60% of the ulcers had reduced in size by 50%. At 8 weeks, 90% of the ulcers were reduced in size, and 30% were completely healed. Therefore, these allografts may be a viable treatment option for category II-III PUs. Similar results were described by Dehghani M et al. in 2017 in Iran, in which the healing of PUs treated with cryopreserved human amniotic membrane allografts was compared with standard care in 24 patients with category II and III PUs. The patients had symmetrical ulcers on the upper and lower extremities. Participants were divided into two groups: amnion and control. The first sign of response, a decrease in wound discharge, was detected 12–14 days after the biological dressing. Complete healing occurred only in the amnion group, with no complications. Partial healing was greater in the amnion group. The healing time in this group was faster than in the control group (20 vs. 54 days). Therefore, the cryopreserved amniotic membrane is an effective biological dressing that promotes reepithelialization in PUs [15,16].

A study conducted a systematic exploratory literature review from 2010 to 2021, including experimental and observational studies, to review various treatments for skin burns, focusing on the use of human amniotic membranes (HAMs). The findings suggest that HAMs can serve as a biological scaffold, promoting faster healing and reducing complications such as infections and hypertrophic scarring [17]. The review highlights several key studies: Mohammadi et al. (2013) [18] performed a randomized controlled trial and discovered that HAM-covered skin grafts had a higher success rate and faster healing compared to traditional methods; Puyana et al. (2019) [19] conducted a retrospective review of pediatric patients with facial burns and found that dehydrated HAMs had fewer complications than cadaveric allografts and Vaheb et al. (2020) [20] carried out a double-blind randomized controlled trial, which indicated that while HAM was not superior to vaseline gauze in healing time, it did improve epithelialization and pain reduction. Overall, HAM is a viable and effective treatment option for partial-thickness burns, offering benefits such as reduced healing time, lower infection rates, and improved pain management

Likewise, they have been used in the reconstruction of the duramater in cranioencephalic trauma, prevention of meningocerebral adhesions, and treatment of premature rupture of hAMs and vaginoplasties [21,22].

The purpose of the present study was to carry out a standardized, easy, and economical protocol for the procurement, processing, and storage of hAMs without affecting structure, ensuring the safety profile of the tissue. We would like to use hAMs for the treatment of PS in hospitalized patients in health institutions in Colombia.

## 2. Materials and Methods

### 2.1. Obtaining and Transporting of Placentas

The placentas were obtained at the La Samarita University Hospital (HUS) in Zipaquirá, Cundinamarca, Colombia. This study was approved by the HUS Hospital’s ethics committee, with record number 11-2020 (Supplementary file S1). The processing and storage of the hAMs were carried out in the Research Laboratory of the University of La Sabana. The criteria for the selection of placenta donors were those established for tissue donors in our country, in accordance with the standards of resolution 5108 of 2005 [23], which establishes the manual of good practices for tissue banks and bone marrow in Colombia.

The exclusion criteria were uncontrolled pregnancy, acute or chronic maternal infection, acute or chronic fetal infection, infection and/or inflammation of the fetal membranes, tattoos or piercings performed in a period of less than six months, sexual promiscuity, mother with physical or mental disability and pregnant women under 18 years of age.

The obstetrician carried out the mother’s prenatal check-ups and verified that the frequency was not less than 3 prenatal visits. Likewise, compliance with the inclusion criteria was verified, and those women who accepted the donation of the placenta and gave their written consent were enrolled in the study (Supplementary file S2). This research complies with the principles of the Declaration of Helsinki. The placenta was obtained by the obstetrician only in cesarean deliveries to avoid bacterial contamination [24].

In the operating room, the placenta was placed in a sterile bag with 300 mL of cold sterile physiological solution (SSN) with antibiotics-antimycotics (10,000 units/mL penicillin, 10,000 µg/mL of streptomycin and 25 µg/mL amphotericin B. Gibco). The bag was placed in a second sterile bag, which was also sealed, and finally, the material was placed in a sterile plastic container with an airtight lid (both the first bag and the container were marked with the identification information of the patient: name of the donor, type and identification number, age in years and data on the place, date, and time of placenta collection). This container was placed in a refrigerator that kept the temperature between 2 and 8 °C, for a maximum of two hours prior to processing.

During the cesarean section, 5 mL of maternal blood was extracted in a tube without anticoagulant to obtain serum and stored in the refrigerator along with the placenta to maintain the cold chain. Once the refrigerator entered the research laboratory of the University of La Sabana (Medical Research Center), the process of obtaining the amniotic membrane began and the blood was sent to the clinical laboratory where the analyses for HIV I and II (Bio-rad^®^ Laboratories Inc., Hercules, Hercules, CA, USA), hepatitis A and B, C, (Abbot^®^, Chicago, IL, USA) antibodies against Trypanosoma cruzi, Toxoplasma IgG and IgM (VirClia^®^, Vircell Microbiologist, Granada, Spain), and hemoclassification were carried out. No more than 8 h should pass from obtaining the placenta in the operating room and processing it in the laboratory.

### 2.2. Processing of Amniotic Membranes

In the biological safety cabinet, the bags containing the placenta were opened and samples of the solution in which it was transported were used to carry out microbiological controls (for aerobic, anaerobic bacteria and fungi). A microbiological culture was performed on 5% sheep blood agar and 5% CO_2_ chocolate agar plates. The samples were incubated at 35 °C for 48 h. For anaerobic microorganisms, Thioglycolate broth was used as a culture medium at 35 °C for 5 days. The samples were then plated on SNVS and SCS agar (BioMérieux, Marcy-l’Etoile, France) and incubated in an anaerobic jar at 35 °C for 7 days. For fungi, the samples were incubated on Sabouraud agar for 7 days. Results were read every 24 h.

**Figure 1:**
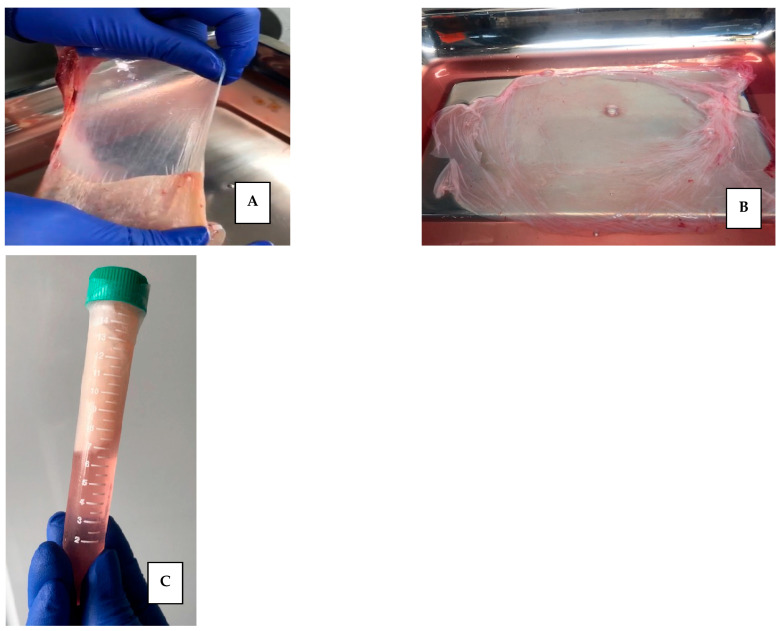
(**A**) Separation of the amniotic membrane from the chorionic membrane. (**B**) Complete hAM before fractionation. (**C**) hAM on nitrocellulose paper preserved in DMEM and glycerol solution at 50% *v*/*v*.

The first step of processing was the separation of the hAM from the chorionic membrane. This process was carried out manually and within the class II A2 biological safety cabinet, as shown in Figure 1A.

The second step began with the washing: the hAMs were washed by shaking in a solution (200 mL) composed of 0.9% SSN and a 50% *v*/*v* antibiotic-antimycotic solution (10,000 units/mL of penicillin, 10,000 µg/mL of streptomycin and 25 µg/mL of amphotericin B. Gibco^TM^,Thermo Fisher Scientific Inc., Waltham, MA, USA) for 3 min. This washing was repeated three times, changing the mixture in each wash. Subsequently, the hAMs were spread on a sterile field and visible tissue debris, blood, and clots were manually cleaned. The shaking washing process was repeated using exclusively sterile SSN at room temperature on three occasions to leave it clean and keep it moist to ensure its integrity. At this point, the placenta was examined macroscopically, and visible anomalies were excluded, and the integrity of the tissue was ensured. See Figure 1B.

The third step was the fractionation of the membrane: hAMs were spread on a surgical tray and sectioned into pieces that were from 2 × 2 cm to 10 × 10 cm, depending on the quality and extension of the membranes, as well as the use that would be given to them. The fragments were placed on a support; we used nitrocellulose paper (Sartorius^®^, Gotinga, Germany), considering placing the epithelial side upward and the stromal surface in contact with the support, with the aim of facilitating its subsequent application [25] Figure 1C.

### 2.3. Storage of Human Amniotic Membranes

Once the hAM was spread on the nitrocellulose paper, it was rolled and introduced into 15 mL Falcon tube containing 12 mL of the freezing solution composed of 50% DMEM Low Glucose (Dulbecco’s Modified Eagle Medium, Capricorn Scientific, Ebsdorfergrund, Alemania) and 50% Glycerol (Thermo Fisher Scientific Inc., Waltham, MA, USA). See Figure 1C. During this step, a sample of the freezing solution (1 mL) was taken to carry out microbiological controls and a tissue sample was taken to confirm the preservation of the tissue structure through histological study. The microbiological studies carried out included the cultivation of aerobic bacteria by mass sowing on blood, chocolate, and MacConkey agars (Microgen LabG&M, Bogotá, Colombia) at 37 °C for 37–48 h of incubation and for anaerobes on MacConkey agar (Microgen^®^) using AnaeroGenTM bags (Thermo Scientific Oxoid™, Waltham, MA, USA) at 37 °C for 37 to 48 h of incubation. The hAMs remained stored at −20 °C.

The membranes remained frozen until the mechanical and histopathological studies started. The Ham was placed in a refrigerator at 4 °C for 6 h to thaw and was then extracted under a laminar flow hood for fixation in formalin (for 24 h) for histological studies. The Ham-nitrocellulose paper pair contained in the Falcon tube was transported in a portable cooler at 4 °C to the mechanical testing laboratory at the Universidad de La Sabana facilities.

### 2.4. Histological Characterization of the hAMs

Fragments of fresh hAMs were fixed in a 10% buffered formalin solution (OneLab^®^, manufacturer, San Diego, CA, USA) for 24 h. At the end of this period, processing was carried out using conventional histotechnique and two slides were stained: one with hematoxylin eosin (H&E) (ACROS ORGANICS^®^, Thermo Scientific, Waltham, MA, USA) and the second with trypan blue (TB) (Sigma^®^, Thermo Scientific, Waltham, MA, USA). This process of fixation, histological processing and staining were repeated with fragments of frozen hAMs at −20 °C.

Histological evaluation was performed using an Axio Scope A1 microscope (LEICA^®^, Wetzlar, Germany) and photographs were taken using the AxioCam IC5 camera (Zeiss^®^, Jena, Germany) with a 40× objective in triplicate (both epithelium and stroma) of six hAMs, three fresh and three hAMs freezing solution.

H&E staining allowed us to evaluate the presence of epithelium, stroma, and cellular characteristics of the AEC to rule out injury and cell death. The integrity criteria described by M, Wagner et al. [26] were applied, which qualifies the epithelium according to its continuity and the clear differentiation of the basement membrane in 4 categories with a score from 0 to 3, with 0 normal histology and 3 a tissue impossible to evaluate given the complete loss of tissue differentiation. Similarly, the integrity of the stroma is classified into 4 categories with a score from 0 to 3, with 0 a stroma composed of continuous, lamellar collagen fibers and 3 the complete disruption of the tissue.

The percentage of viability of AEC of each of the six hAMs stained with H&E was determined by testing 100 cells in three different fields of high power (40×) and calculating its average and standard deviation. Criteria for cell injury/death included intense cytoplasmic eosinophilia, nuclear irregularities, alterations in chromatin distribution, karyolysis, karyorrhexis or karyopyknosis, and rupture of the cytoplasmic membrane. Additionally, TB staining was performed as a second test to determine viability, following the same evaluation criteria as for H&E staining.

### 2.5. Mechanical Properties of hAMs

The mechanical behavior of hAMs is investigated through tests measuring the membranes’ ability to elongate when subjected to a uniaxial tensile force. The tensile strength describes the ability of hAMs to resist tension and is determined as the maximum stress point observed before rupture. The modulus of elasticity describes the stress–strain relationship and characterizes the stiffness of hAMs. This modulus is determined as the slope of the initial portion of the stress–strain curve. Maximum deformation gives the idea of how much ductility the material has; the final value of deformation is considered the property.

In this study, the mechanical properties of three fresh hAMs and three previously frozen hAMs were determined through uniaxial tension tests. The dimensions of the hAMs used were 20 mm × 100 mm, and their thickness was measured using a micrometer gauge (Rexbeti, digital micrometer, Auburn, WA, USA). The hAMs were placed in the testing machine (Instron 3026, High Wycombe, Buckinghamshire, UK) equipped with a 5 kN load cell and subjected to a deformation speed of 10 mm/min. The machine’s software recorded both the applied force and the experienced elongation. Tests were stopped upon the observation of a sudden decrease in force, indicating rupture in the hAM.

The force and elongation data obtained were normalized with respect to geometry to obtain a stress–strain curve, allowing for the determination of their properties. Stress is defined as force divided by the cross-sectional area and is measured in units of N/mm^2^. Deformation is the elongation divided by the initial length of the testing specimen.

## 3. Results

The results of the cultures of transportation and freezing solution were negative for aerobic and anaerobic bacteria and fungi, ruling out contamination of the hAMs. Carrying out microbiological controls confirmed the sterility preservation of hAMs when we applied this protocol.

**Figure 2:**
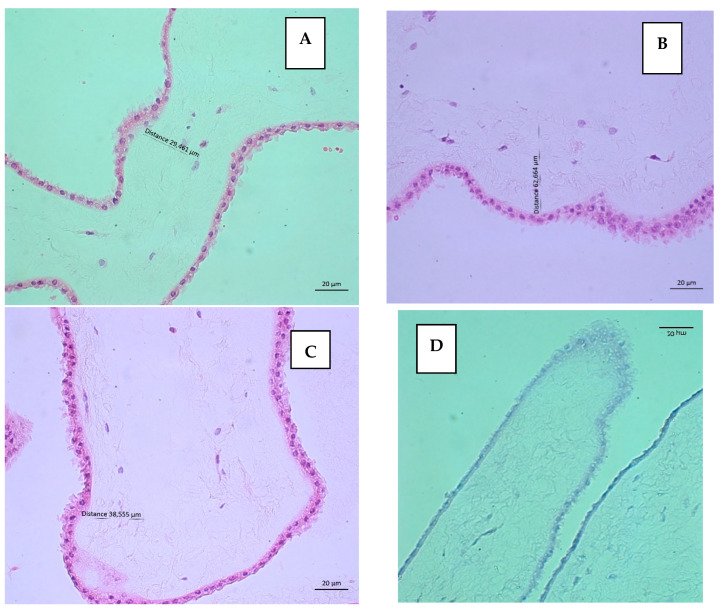
Fresh hAM. (**A**–**C**) These images showed the preservation of tissue architecture, integrity of the epithelium composed of cubic amniotic cells and supported by a well-defined basement membrane with intact connective stroma in one donor. Epithelial score of 0 and stromal score of 0 from integrity evaluation for triplicate in H&E. 40×. (**D**) The nuclear negativity with TB staining allows us to infer that AEC is viable. 40×.

**Figure 3:**
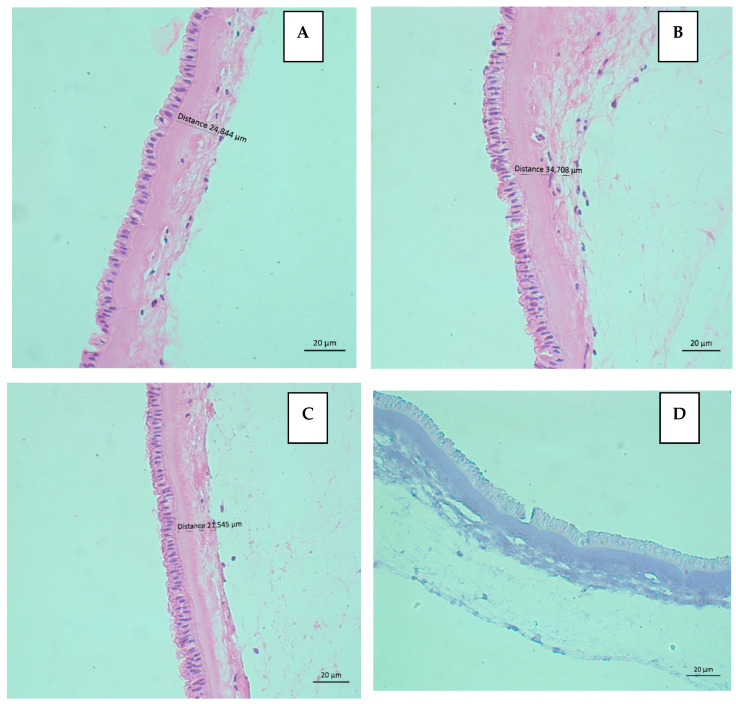
Frozen hAM. (**A**–**C**) Integrity of the epithelium could be observed and some focal disruptions of the stroma were detected in one donor. Epithelial score of 0 and stromal score of 1 from integrity evaluation for triplicate in H&E 40×. (**D**) The nuclear negativity with TB staining allows us to infer that AEC is viable. 40×.

The histological study showed hAMs composed of both epithelium and intact stroma with adequate preservation of the structure. The integrity score results are in Table 1. Table 2 presents the results of the viability evaluation of the AECs with H&E stain. Additionally, TB staining was negative in all nuclei of AECs of both types of hAMs, demonstrating viability. Figure 2 shows representative images of the histological evaluation of one fresh hAM and Figure 3 from one frozen hAM.

**Figure 4:**
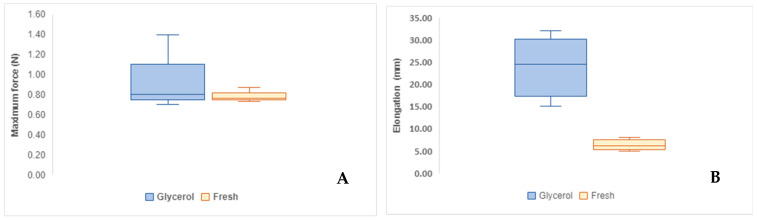
(**A**) Comparison of the maximum force resisted by fresh and Glycerol-frozen hAM. (**B**) Comparison of the elongation resisted by fresh and Glycerol-frozen hAM.

In Figure 4A, a comparison of the maximum force reached by each membrane group is presented. HAMs exposed to glycerol exhibited a maximum force of Fmax=0.97±0.378 N, while fresh hAMs resisted a Fmax = 0.79 ± 0.073 N. In Figure 4B, an elongation of ΔL = 24 ± 8.54 mm is observed for hAMs exposed to glycerol, compared to ΔL = 6.33 ± 1.52 mm for fresh hAMs.

**Figure 5:**
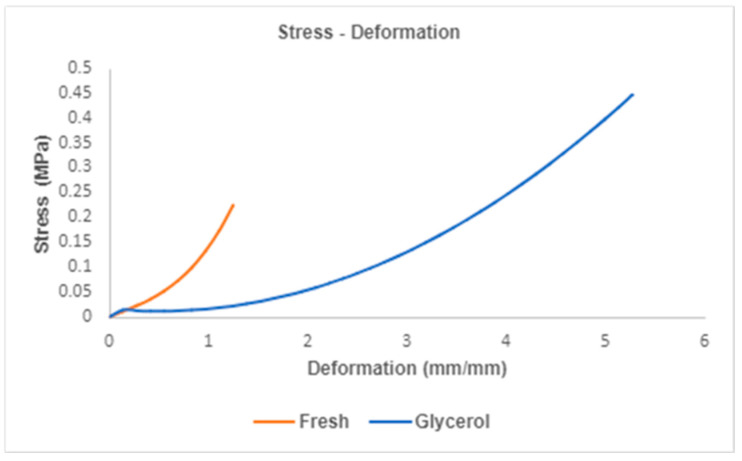
Stress vs. Strain Results. From this graph, the mechanical properties of maximum stress, deformation, and elastic modules are derived.

Figure 5 illustrates the stress–strain behavior for both fresh and glycerol-treated hAMs, derived from tensile testing. The graph highlights the non-linear mechanical response typical of biological membranes. The stress–strain curves reveal that while glycerol treatment does not significantly affect the stress–resistance capability of the membranes (*p* > 0.05), it has a pronounced impact on their deformation capacity (*p* < 0.05), as confirmed by ANOVA analysis in Table 3.

From these results, the maximum stress and deformation for each membrane type were derived. ANOVA results indicate no significant difference in maximum stress between the groups, with fresh hAMs exhibiting 0.779 ± 0.665 MPa and glycerol-treated hAMs achieving a similar value of 0.779 ± 0.665 MPa (*p* = 0.563). However, the deformation capacity differed significantly, with glycerol-treated hAMs achieving an average strain of 2.4 mm/mm compared to 0.633 mm/mm for fresh hAMs (*p* = 0.024).

Additionally, ANOVA revealed a significant reduction in the elastic modulus for glycerol-treated membranes E = 2.3 Pa compared to fresh membranes E = 120.2 Pa (*p* = 0.0031). This marked reduction in stiffness suggests that glycerol treatment substantially alters the membrane’s mechanical properties, enhancing its flexibility and deformation capacity.

The investigation showed glycerol’s impact on the mechanical properties of hAMs and revealed significant insights with implications for tissue engineering and regenerative medicine. While glycerol appears to offer benefits in terms of membrane preservation and adaptability, it also prompts a reevaluation of how these mechanical changes influence grafting success and long-term clinical outcomes. A limitation of our study is that we only evaluated hAM samples for up to one week frozen at 20 °C.

## 4. Discussion

hAMs have been used for tissue engineering and regenerative medicine applications due to biocompatibility, antibacterial activity, immunomodulatory effects and ability to promote healing and anti-scarring processes. However, the use of the hAM is subject to the generation of a protocol that guarantees the quality of the future implant. In this article we present a protocol that meets these characteristics.

The hAMs were collected from consenting mothers following elective cesarean-section delivery under sterile conditions, as recommended by Ingraldi et al., who refers to “…vaginal flora contributes to an increased bioburden and a decrease in the tensile strength of the membranes associated with epithelial to mesenchymal transition (EMT) during labor” [27].

In the United States, the Center for Biologics Evaluation and Research (CBER) regulates tissue, cellular, and tissue-based products, and it outlines the regulations for minimizing disease transmission. This center recommends taking a blood sample from the placenta donors for serology, including human immunodeficiency virus types I and II, human T-cell lymphotropic virus, human hepatitis virus types B and C, and syphilis. Additional testing may be performed for cytomegalovirus (CMV), toxoplasma, tuberculosis, Creutzfeldt-Jakob disease, or other infections per regulatory guidelines [28].

In Colombia, The Ministry of Health and Social Protection through the Decree number 2493 of August of 2004—partially regulating Laws 9 of 1979 and 73 of 1988 concerning anatomical components—stipulates in Chapter IV /Art 18 that tissue banks and health service providers are required to perform the following tests on all donors under their care: serological test for syphilis: detection of the surface antigen of the Hepatitis B virus (HBsAg), detection of antibodies against Hepatitis C virus, Human T-cell lymphotropic virus (HTLV 1 and 2), HIV 1–2, Trypanosoma Cruzii, CMV and Epstein Baar virus (EBV) [29].

Additionally, in Colombia, the regulation of hAM is governed by Resolution 5108 of 2005, issued by the Ministry of Health and Social Protection, which establishes a comprehensive framework for tissue banks, including those handling amniotic membranes. To operate, these banks must obtain a Certificate of Compliance with Good Practices from Invima (Instituto Nacional de Vigilancia de Medicamentos y Alimentos), which is renewed periodically and requires annual inspections.

The regulation mandates the implementation of a Quality Management System, ensuring the safety and viability of tissues through internal and external audits. Donors of amniotic membranes must be healthy mothers with controlled pregnancies and no transmissible infections, and they must provide informed consent.

The processing and preservation of amniotic membranes involves methods like cryopreservation and lyophilization, with strict microbiological and anatomical quality controls. Each unit of the amniotic membrane must be clearly labeled to ensure traceability from donation to final use, including information on tissue type and storage conditions [23].

Following these standards, this protocol included the collection of a maternal blood sample at the time of cesarean for microbiological assays of seven microorganisms, which were negative in all cases. However, CMV and EVB are not included in this panel, because it is determined whether health insurance covers it or the patient pays for it.

The next critical step is the transport of the material from the hospital to the laboratory and its transfer to the clean room [30]. The literature review conducted by Leal-Marin S. et al. states that if the transportation time is less than 2 h after the delivery, the tissue may be transported at room temperature; however, if the time is longer than two hours, it should be performed at 2–8 °C [31]. Our protocol maintains the cold chain in the suggested temperature range independent of the extraction time. Additionally, the transport protocol, described in Section 2.1, also maintains sterile conditions, which is corroborated by the microbiological results obtained from the evaluation of transport medium.

Various transport medium options are found in the literature: Gholipourmalekabadi et al. refer to the widespread use of a mixture composed of Eagles’ minimum essential medium supplemented with L-glutamine, antibiotic cocktail (gentamicin, penicillin, and ciprofloxacin) and Amphotericin B, while Leal-Marin S. et al. refer to the use of sterile solution, such as Phosphate-Buffered Saline, placed in the container to protect the tissue’s dehydration [30,31]. Our protocol uses SSN with antibiotics and antifungal agents, which are cheaper and effective for supporting sterility conditions.

The use of 50% glycerol is a standard method for the preservation of hAMs for tissue engineering and regenerative applications in the European Union [26]. The mixture of DMEM and 50% *v*/*v* glycerol is suitable as a freezing solution, which has already been referred to in the scientific literature [32,33]. We decided to use a freezing solution with this composition; however, Ravishanker R et al. demonstrated that the hAM frozen in 85% glycerol in SSN at 80 °C for 2.5 years is effective in the treatment of superficial and superficial partial thickness burns. These findings make us think about the possibility of increasing the concentration of glycerol to extend the use time of the hAMs [34].

The evaluation of cell viability was carried out initially by TB staining due to its low cost and ease of execution. This procedure is the same that is used to evaluate the viability of cells in corneas in many eye tissues banks [35,36]. This stain demonstrates the absence of nuclear staining of AEC in fresh and frozen hAMs. Also, a quantification of viable AEC with H&E found a high percentage of viability for fresh and frozen membranes was carried out.

These results confirm the ability of the preservative solution to avoid cell death, which is in line with what was reported by Maral T et al. [37], who demonstrated that glycerol can be used as a preservative effectively with the additional advantage of having an antiviral and antibacterial effect, thus guaranteeing the sterility. The preservation of AEC guarantees the production of bFGF [26] (and other GF and components of the basement membrane) and ensures the availability of the biomolecules that produce the effect of cell proliferation and migration.

The cell membrane of AEC allows small particles such as glycerol (MW 92 g/mol), the preservative solution that we used in our study, to pass through the plasma membrane, allowing its entry into the cytoplasm and the osmotic replacement of intracellular water during the frozen process, which explains a higher percentage of viable AEC in frozen hAMs [38,39]. This preservative solution also increases the volume of water in the extracellular medium and absorbs moisture from the surrounding medium; therefore, it is lubricating and humectant. The observation of clear spaces in the depth of the stroma of frozen hAMs suggests tissue expansion due to greater attraction and retention of water by glycerol, which can easily diffuse through the different layers of hAMs. Due to the composition of the spongy layer, which presents a greater proportion of hydrophilic non-fibrillar proteins, the increase in water accumulation in this area could be explained [39].

The potential influence of glycerol on the mechanical properties of hAM is of critical importance for grafting practices and clinical applications. Jahanafrooz Z et al. showed that storage methods (with glycerol additive) do not affect the tensile strength and Young’s modulus of hAM [40], but our results showed modifications in frozen membranes: increased deformation capacity and reduced elastic modulus. These changes may enhance the membrane’s adaptability and conformability to diverse wound surfaces, potentially improving the healing process.

Previous studies indicate that the composition of hAMs also determines their mechanical behavior [38]. There are studies demonstrating how the amount, organization, and direction of collagen fibers in the extracellular matrix affect the ability to resist tensile forces [41]. The elastic modulus has been shown to be associated with the content of elastin fibers, laminin, and even hyaluronic acid [42]. Likewise, the hydration process observed in the hAMs could potentially elucidate the alterations in elasticity noted in hAMs preserved in glycerol, as supported by the modulus of elasticity outcomes. Finally, Jahanafrooz Z, et al. state that tensile strength and Young’s modulus increased with longer storage time [40].

Nevertheless, the existing gap in literature on the direct correlation between these mechanical alterations and clinical outcomes underscores the need for further research to fully understand the implications of glycerol-treated hAMs in regenerative medicine. This discussion emphasizes the complex balance between preserving hAMs’ beneficial properties through cryopreservation and maintaining their mechanical integrity for successful clinical use. Further research and clinical studies are crucial to bridge the knowledge gap, ensuring that hAMs remain an effective option for tissue regeneration.

The cost of processing and preservation of the hAMs is lower compared to other tissue/cell-engineered skin substitutes available on the market. As demonstrated by Glat P et al., the treatment of 60 patients with diabetic foot ulcers assigned to two treatment groups—dehydrated human amnion and chorion allograft (dHACA) vs. tissue-engineered skin substitute (TESS)—with a 12-week follow-up, showed the mean product cost for the dHACA was $2200 (median: $1300) while for TESS it was $7900 (median: $6500)]. The study concluded that the cost of healing diabetic foot ulcers using dHACA is significantly lower. Additionally, the use of hAMs avoids the process of manufacturing [43].

Likewise, the use of autologous grafts generates morbidity in the tissue donor area, and heterologous grafts have the potential for immunological rejection. This aspect is another advantage of the use of hAMs, which have a low or absent level of expression of HLA class I molecules and absence of HLA class II molecules, thus avoiding allograft rejection.

## 5. Conclusions

Our protocol allows obtaining sterile hAMs with adequate preservation of the AEC and the use of the nitrocellulose membrane, guaranteeing adequate positioning of the hAMs at the time of their use as a skin implant.

The findings highlight the necessity of developing standardized protocols for functional integrity evaluation because the use of glycerol as a preservative modifies the mechanical proprieties of the hAMs and it is necessary to repeat microbiological, cell viability and mechanical studies on the frozen hAMs at 6 and 12 months to ensure that long-term storage conditions do not affect the quality.

We agree with other authors about including additional consent from the mother for subsequent screening of HIV at 3–4 months post placenta collection and preserve the hAMs for 6 months until confirming negative screening result for HIV prior to use for transplant. We consider it necessary to test for CMV and tuberculosis.

The application of hAMs has demonstrated efficacy and safety in the treatment of vascular ulcers and diabetic ulcers; however, there are not enough studies on its usefulness in the treatment of PUs. We consider that generating a protocol for obtaining, processing, and preserving hAMs is the first step to promoting research into the potential use of these natural scaffolds as a strategy for the treatment of non-healing PUs.

## Figures and Tables

**Table 1:** Results of the application of the integrity score to evaluate the histology of hAMs.

Human Amniotic Membranes *		Integrity Score			
		Epithelium		Stroma	
		Value	Average (SD)	Value	Average (SD)
1	Field 1	1	0.67 SD (0.47)	0	0.33 SD (0.47)
	Field 2	1		0	
	Field 3	0		1	
2	Field 1	1	0.33 SD (0.47)	0	0 SD (0)
	Field 2	0		0	
	Field 3	0		0	
3	Field 1	0	0 SD (0)	0	0 SD (0)
	Field 2	0		0	
	Field 3	0		0	
4	Field 1	1	0.67 SD (0.47)	0	0.33 SD (0.47)
	Field 2	0		1	
	Field 3	1		0	
5	Field 1	0	0 SD (0)	1	1 SD (0)
	Field 2	0		1	
	Field 3	0		1	
6	Field 1	0	0.67 SD (0.47)	0	0.67 SD (0.47)
	Field 2	1		1	
	Field 3	1		1	

* Numbers 1–3 fresh hAMs and 4–6 frozen hAMs. SD: standard deviation.

**Table 2:** Results of the viability evaluation of human amniotic membranes using H&E.

		Viable Cells		Injury/Death Cells	
Human Amniotic Membranes *		Count	Average (SD)	Count	Average (SD)
1	Field 1	82	74 SD (6.94)	18	26 SD (6.94)
	Field 2	65		35	
	Field 3	74		26	
2	Field 1	80	83 SD (5.25)	20	17 SD (5.25)
	Field 2	78		22	
	Field 3	90		10	
3	Field 1	85	83 SD (2.36)	15	17 SD (2.36)
	Field 2	80		20	
	Field 3	85		15	
1	Field 1	89	88 SD (1.25)	11	12 SD (1.25)
	Field 2	86		14	
	Field 3	88		12	
2	Field 1	87	87 SD (2.08)	13	13 SD (2.05)
	Field 2	90		10	
	Field 3	85		15	
3	Field 1	88	90 SD (1.63)	12	10 SD (1.63)
	Field 2	90		10	
	Field 3	92		8	

* Numbers 1–3 fresh hAMs and 4–6 frozen hAMs. SD: standard deviation.

**Table 3:** ANOVA analysis for the mechanical properties: Maximum stress, maximum deformation and elastic modulus.

ANOVA Analysis (*p* = 0.05)			
	Glycerol	Fresh	Probability
Stress (Mpa)	0.779	0.665	0.563
Deformation (mm/mm)	0.63	2.4	0.024
Elastic modulus (Pa)	2.3	120.2	0.0031

## Data Availability

This research work does not correspond to a clinical trial. All data are reported in the article.

## Supplementary Materials

The following supporting information can be downloaded at: https://www.mdpi.com/article/10.3390/mps8010012/s1, File S1: Approval of the hospital’s ethics committee; File S2: Informed consent.
