# Supplementary material for: Procurement, Processing, and Storage of Human Amniotic Membranes for Implantation Purposes in Non-Healing Pressure Ulcers
Source: Methods Protoc. 2025 Feb 1;8(1):12. doi: 10.3390/mps8010012 (PMC11858804; doi:10.3390/mps8010012)
Supplement: Supplementary file 1 [file mps-08-00012-s001.zip › mps-3229679-Supplementary file S1.pdf]

Bogotá D.C., 26 de Noviembre del 2020

Respetados Investigadores

Lina Andrea Gómez Restrepo

Carlos Andrés Domínguez Paz

Andrea Milena García Becerra

Juan Fernando Ospina

Edwin Alfonso Cruz

Grupo Investigación:

Grupo de Investigación Biomédica Universidad de La Sabana (CIBUS)

Grupo de Investigación en Energía, Materiales y Ambiente (GEMA)

Grupo de Investigación de Ginecología del Hospital Universitario de la Samaritana (GINECOHUS)

Cordial saludo,

En reunión el 19 de Noviembre del 2020 ante el Comité de Ética en Investigación del Hospital Universitario de La Samaritana (CIEHUS), realizado en forma virtual a través de la plataforma Google Meet, según Acta de Reunión No. 11-2020 llevada a cabo a las 10:13 a. m. se presentó el proyecto del grupo de investigación GINECOHUS titulado

"Evaluación de dos métodos de descelularización de membranas amnióticas humanas para su uso en ingeniería de tejidos".

Luego del análisis, consulta y discusión de los aspectos científicos, metodológicos y éticos del proyecto por parte de los miembros del comité, se decidió por unanimidad que el proyecto es:

APROBADO *SIN MODIFICACIONES*.

Atentamente,

Amparo Gómez Gélvez

Presidente del Comité

Proyectado por: Amparo Gómez Gélvez
