# Supplementary material for: Procurement, Processing, and Storage of Human Amniotic Membranes for Implantation Purposes in Non-Healing Pressure Ulcers
Source: Methods Protoc. 2025 Feb 1;8(1):12. doi: 10.3390/mps8010012 (PMC11858804; doi:10.3390/mps8010012)
Supplement: Supplementary file 1 [file mps-08-00012-s001.zip › mps-3229679-Supplementary file S2.pdf]

		CODIGO:	
	**CONSENTIMIENTO INFORMADO PARA DONACIÓN DE PLACENTA HUMANA**	VERSION:	1
		FECHA:	

Título del proyecto:

Evaluación de dos métodos de descelularización de membranas amnióticas humanas para su uso en ingeniería de tejidos

Investigador Principal: Lina A. Gómez Restrepo. Cel: 3113303448

Objetivo y beneficios esperables:

La membrana amniótica, procedente de la placenta, es un tejido que protege al feto y brinda un medio adecuado para su crecimiento. Una vez nace el bebé, ya no es necesario ni para el bebé, ni para la madre y son descartados en la basura.

Después de la donación de una placenta, por parte de una madre que acaba de tener a su hijo(a) por cesárea, se somete a un procesamiento que permite ser utilizada como tratamiento en alteraciones de la piel y de las corneas.

El Objetivo Principal del estudio es evaluar dos técnicas de preparación de membranas amnióticas humanas descelularizadas y su efecto sobre sus propiedades bioquímicas y estructurales.

Descripción del procedimiento: Usted ha cumplido con cinco controles prenatales normales y sus resultados de laboratorio cumplen con los criterios de inclusión para el presente estudio.

Si usted realiza la donación, luego de la cirugía (cesárea), el equipo médico que la atiende entregará la placenta al investigador responsable de trasladarla al Medical Research Center de la Universidad de la Sabana para obtener las membranas amnióticas y realizar los

ensayos propuestos en el presente estudio. Con los resultados de este estudio, sabremos si el tratamiento propuesto para las membranas amnióticas, le provén características para ser usadas en el futuro como tratamiento para pacientes con alteraciones tisulares.

El Dr. Juan Fernando Ospina, ginecobstetra del Hospital de la Samaritana de Zipaquirá, revisará los resultados de sus controles prenatales y guardará la confidencialidad de los datos obtenidos y de la identidad de los pacientes.

Duración de la participación en el estudio: su participación será una única vez, con la donación de una placenta.

Alternativas disponibles: Usted podría optar por no realizar la donación de la membrana amniótica, no tendrá ninguna consecuencia negativa para usted o su hijo(a) si toma esa decisión.

Consecuencias previsibles: El proceso de donación no produce daños o efectos secundarios sobre usted o su hijo(a). Las placentas son tejidos que son descartados después de la cesárea, no tendrán efectos secundarios o reacciones adversas en quien las done. Los pacientes que acepten donar su placenta para este estudio no tendrán ningún riesgo por la donación y esperamos que los resultados de este estudio puedan beneficiar a muchos pacientes que sufren alteraciones tisulares y puedan en un futuro ser tratados con membranas amnióticas.

Riesgos: En el caso de la donación de membrana amniótica, al tratarse de un tejido que ni el bebé ni la madre necesitan después de la cesárea, el proceso de donación no produce daños o efectos secundarios indeseables. Se garantiza que la participación de las madres es voluntaria y no tiene ninguna responsabilidad en la investigación.

Información de interés: Los pacientes tienen la posibilidad de retirarse libremente y en cualquier momento sin desmedro de la calidad en la atención que se les preste. Los tejidos donados serán usados solamente para fines de investigación.

Compromiso de los investigadores: Los investigadores nos comprometemos a publicar los resultados derivados de esta investigación, esperando que se beneficien pacientes que sufran alteraciones tisulares.

Acepto que he entendido la información que se me ha dado por parte del personal médico y/o investigador principal del proyecto de investigación.

En la Ciudad de Zipaquirá, a los______ días, del mes de ________________ del año _________, YO ____ con domicilio en la ciudad de_________________________ , en pleno uso de mis facultades mentales y libre de toda coacción, doy mi consentimiento para la donación de la placenta, cuyo fin será la utilización con fines de investigación.

Nombre, documento de identificación y Firma de la donante

Nombre: ____

Documento de identificación: _____________________________

Firma del donante: ______________________________________

Nombre, documento de identificación del Testigo

Nombre: ______________________________________

Documento de identificación: ______________________________

Firma del testigo: ____

Nombre, documento de identificación del médico hospitalario

Nombre: ______________________________________

Documento de identificación: ______________________________

Firma del médico: ______________________________________
